# Enhanced Photocatalytic Degradation of Methylene Blue Using Ti-Doped ZnO Nanoparticles Synthesized by Rapid Combustion

**DOI:** 10.3390/toxics11010033

**Published:** 2022-12-29

**Authors:** Sutthipoj Wongrerkdee, Sawitree Wongrerkdee, Chatdanai Boonruang, Supphadate Sujinnapram

**Affiliations:** 1 Department of Physics, Faculty of Liberal Arts and Science, Kasetsart University Kamphaeng Saen Campus, Kamphaeng Saen, Nakhon Pathom 73140, Thailand; 2 Faculty of Engineering, Rajamangala University of Technology Lanna Tak, Muang, Tak 63000, Thailand; 3 Department of Physics and Materials Science, Faculty of Science, Chiang Mai University, Chiang Mai 50200, Thailand; 4 Center of Excellence in Materials Science and Technology, Chiang Mai University, Chiang Mai 50200, Thailand

**Keywords:** ZnO, titanium, combustion, photocatalyst, surface area, methylene blue

## Abstract

ZnO and Ti-doped ZnO (Ti-ZnO) nanoparticles were synthesized using rapid combustion. The morphology of ZnO and Ti-ZnO featured nanoparticles within cluster-like structures. The ZnO and Ti-ZnO structures exhibited similar hexagonal wurtzite structures and crystal sizes. This behavior occurred because Zn^2+^ sites of the ZnO lattice were substituted by Ti^4+^ ions. The chemical structure characterization implied the major vibration of the ZnO structure. The physisorption analysis showed similar mesoporous and non-rigid aggregation structures for ZnO and Ti-ZnO using N_2_ adsorption–desorption. However, Ti-ZnO demonstrated a specific surface area two times higher than that of ZnO. This was a major factor in improving the photocatalytic degradation of methylene blue (MB). The photocatalytic degradation analysis showed a kinetic degradation rate constant of 2.54 × 10^−3^ min^−1^ for Ti-ZnO, which was almost 80% higher than that of ZnO (1.40 × 10^−3^ min^−1^). The transformation mechanism of MB molecules into other products, including carbon dioxide, aldehyde, and sulfate ions, was also examined.

## 1. Introduction

Due to the expansion of the industrial sector and an increase in pollution over the past few decades, a wide range of pollutants have been released and accumulated in environmental systems, especially water. Wastewater and the treatment of complex contaminations in wastewater are major environmental concerns. Moreover, synthetic dyes are modern contaminants in wastewater. Dye molecules are typically designed to be environmentally stable, complex, and non-biodegradable, making them difficult to degrade or remove [1,2]. One of the interesting wastewater treatment strategies is photocatalytic degradation, a low-cost and effective method for pollutant transformation [3,4,5,6,7]. The photocatalytic degradation process requires semiconducting materials to act as a photocatalyst, which can be activated by absorbing the irradiated photons with an appropriate wavelength. After absorbing the photon energy by the photocatalyst, the electrons in the valence band (VB) are excited and promoted to the conduction band (CB), leaving holes at VB [8]. The generated electron-hole (e^−^-h^+^) carriers continuously migrate to the surface to interact with H_2_O/O_2_, inducing ${\mathrm{OH}}^{\cdot -}$/$O_{2}^{\cdot -}$ radicals. The radicals then interact and transform dye molecules into other phases, such as minerals and CO_2_ [9,10].

ZnO is one of the popular semiconductors used as an alternative photocatalyst for effective wastewater treatment [11]. Moreover, ZnO is non-toxic, chemically and optically stable, biocompatible, and cheap [12]. However, the low surface area of ZnO, compared with TiO_2_ [2,8], is an important issue because the surface area is an essential factor directly affecting photocatalytic performance [10], as chemical reactions of pollutant molecules occur at the photocatalyst’s surfaces. Nevertheless, the low surface area also decreases the charge transfer affecting the recombination, which diminishes the photocatalytic performance [13]. To overcome this issue, several studies have presented various kinds of strategies to increase the surface area and reduce the recombination of ZnO photocatalysts [14,15,16]. The application of ZnO doped with an alkaline earth metal, such as barium (Ba), was reported as the photocatalyst [14]. The synthesis of Ba-doped ZnO (BaZnO) was performed by facile chemical precipitation using zinc nitrate hexahydrate and barium nitrate. The particle and crystal sizes of BaZnO were almost halved compared with those of pure ZnO. This caused a small increase in the surface area from 13.35 to 15.00 m^2^/g and a larger induced dislocation density from 1.52 × 10^−4^ to 5.88 × 10^−4^ nm^−2^. The photocatalytic activity of BaZnO to degrade carbaryl solution using UV irradiation was also evaluated. BaZnO exhibited a better photocatalytic performance than that of pure ZnO by around 1.4 times. The incorporation of Pd-metal plasmons into ZnO was presented as an effective way to improve the ZnO surface area [15]. The Pd-ZnO nanoparticles (NPs) were synthesized using a green microwave-assisted method from orange peel extract as a capping agent with a Pd addition. The optimum Pd addition resulted in obtaining the maximum surface area of 16.9 m^2^/g for Pd-ZnO NPs in comparison with that of 9.2 m^2^/g for pure ZnO. The increased surface area was correlated to the decreased crystal size, suggesting the role of aggregation prevention for Pd in ZnO crystallization and the decrease in particle sizes. The Pd-ZnO NPs were then used in a photocatalytic process of degrading the reactive yellow 15 organic dye under sunlight illumination. The Pd-ZnO NPs showed excellent photocatalytic degradation performance, better than that of pure ZnO by around 1.9 times due to the increased surface area. Another interesting photocatalyst structure of ZnO/TiO_2_ composites was synthesized using a hydrothermal process with different capping agents, including cetyltrimethylammonium bromide (CTAB), acetylacetone (ACAC), and poly (4-styrene sulfonic acid) (PS) [16]. The composites prepared with ACAC showed a considerable surface area of 217.08 m^2^/g in comparison with that of 14.05 m^2^/g for pure ZnO. This was caused by the small crystal size, similar to the abovementioned study [14,15]. The increased surface area of the ZnO/TiO_2_ structure caused the enhanced photocatalytic degradation of Tartrazine dye aqueous solution, which was almost two times higher than pure ZnO. The enhancement was attributed to the high surface area of ZnO/TiO_2_ composites, due to the synergy of ZnO and TiO_2_ NPs. However, it is a complex method for synthesizing composite structures and requires many initial materials, which leads to high-cost production. Therefore, the modification of ZnO material using a simple method was considered in this study. Pure ZnO with a low specific surface area was enhanced using Ti-doping. It was aimed at improving the surface areas of Ti-doped ZnO (Ti-ZnO). Furthermore, the doping effect also caused varieties in the mechanical, electronic, and optical properties of the ZnO host material. Generally, there are several methods for synthesizing ZnO, such as precipitation, sol-gel, hydrothermal method, electrochemical process, and combustion [17,18]. In this study, rapid combustion was used to synthesize ZnO and Ti-ZnO because of its simplicity and short time process. The synthesized ZnO and Ti-ZnO were then characterized by several techniques and utilized as photocatalysts for degrading methylene blue (MB) in water.

## 2. Materials and Methods

**Figure 1:**
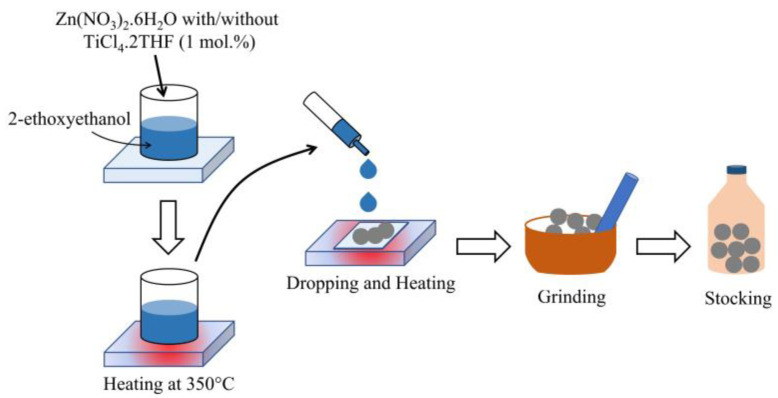
The illustrated flow diagrams of ZnO and Ti-ZnO NPs synthesized by rapid combustion.

ZnO was synthesized by the rapid combustion technique. For the preparation of the Zn source, zinc nitrate hexahydrate (Zn(NO_3_)_2_·6H_2_O) was dissolved in 20 mL of 2-ethoxyethanol (C_4_H_10_O_2_) at the initial concentration of 1 M under continuous stirring at room temperature for 30 min to form a homogenous zinc complex solution. Before combustion, the solution was heated to 350 °C for 10 min. Then, the solution was dropped on heated-glass substrates (350 °C) for the smoldering combustion process. After 20 min, the substrate was left to cool down to room temperature. Next, the obtained white product of ZnO was scratched from the glass substrate, ground for 1 h, and stocked in a brown bottle. A similar process was carried out to synthesize Ti-ZnO using a zinc–titanium complex solution instead of a zinc complex solution. The zinc–titanium complex solution was prepared by co-dissolving zinc nitrate hexahydrate and 1 mol.% titanium chloride tetrahydrofuran complex (TiCl_4_·2THF) in 20 mL of 2-ethoxyethanol. The preparation flow diagram was illustrated in Figure 1.

The synthesized combustion products of ZnO and Ti-ZnO were characterized using several methods. The morphology was observed using a scanning electron microscope (SEM, JSM-6610 LV, JOEL) equipped with an energy dispersive X-ray spectrometer (EDS, INCAx-act, OXFORD) for elemental detection. The high-resolution morphology and selected area electron diffraction (SAED) were investigated using a transmission electron microscope (TEM, TECNAI G^2^ 20 S-TWIN, FEI). The crystal structure was determined by X-ray diffraction (XRD) patterns recorded on an X-ray diffractometer (D8 Advance, Bruker). The crystal sizes of ZnO and Ti-ZnO were calculated using the Debye–Scherrer equation (Equation (1)) [14,19]:

D = kλ/βcosθ(1)

where k and λ are the shape factor of the XRD measurement (0.89) and the wavelength of the X-ray source (1.5406 Å), respectively. The β and θ are the full width at half maximum (FWHM) and the diffraction angle for each plane, respectively. A surface functional group analysis was conducted using Fourier transform infrared spectroscopy (FTIR, Spectrum Two, PerkinElmer). A pore analysis was performed by physisorption of N_2_ adsorption–desorption at 77 K using a Tristar II3020, Micrometrics. The adsorption–desorption isotherm was plotted to identify the pore structure. The pore width and specific surface area were analyzed using the Barrett–Joyner–Halenda (BJH) and Brunauer–Emmett–Teller (BET) methods. The band gap energy (E_g_) was investigated from the diffuse reflectance absorption spectra for ZnO and Ti-ZnO using an ultraviolet-visible-near infrared (UV–Vis-NIR) spectrophotometer (UV-3101 PC, Shimadzu). The recorded spectra were then analyzed according to the Tauc’s relation (Equation (2)) [20,21,22]:

(αhν)^1/n^ = A(hν − E_g_) (2)

where α, A, h, and υ are the optical absorbance coefficient, constant, Planck’s constant, and frequency, respectively. For a direct electron transition material, including ZnO, the parameter n is 1/2. The synthesized ZnO and Ti-ZnO were utilized to degrade the MB solution under UV irradiation and demonstrate their potential as an applicable photocatalyst. The MB solution was prepared by dissolving 5 mg of MB in 1000 mL of DI water. It was then stirred for 1 h in the dark. The amount of 0.1 g of ZnO or Ti-ZnO was added into a separate beaker containing 100 mL of the MB solution. It was then stirred for 15 min to disperse the photocatalyst. Then, the photocatalyst-contained MB solution was left for 30 min in the dark condition to reach an adsorption–desorption equilibrium. The photocatalytic process was then activated by the irradiation of a UV lamp (λ~365 nm, 4.5 W/m^2^) for 150 min. To evaluate the photocatalytic degradation performance, a portion of the degraded MB solution was collected at every 30 min interval. The collected solution was examined by measuring the absorbance to monitor the remaining MB molecules. To analyze the MB degradation performance, the degradation rate constant (k) was estimated using the pseudo-first-order kinetics (Equation (3)) [21,23]:

ln(A/A_0_) = −kt (3)

where A_0_ and A are the absorbances of the MB solution at the initial and interval irradiation times, respectively. The degraded MB solution was further investigated using FTIR to monitor the transformed products.

## 3. Results and Discussion

**Figure 2:**
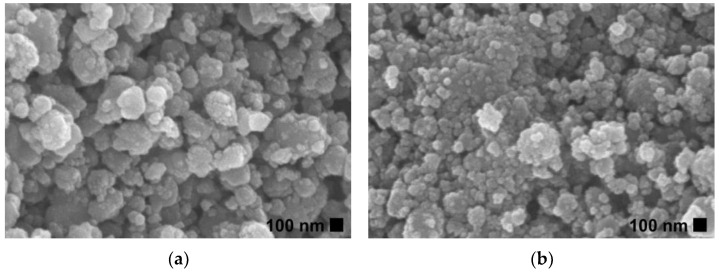
SEM images of (**a**) ZnO and (**b**) Ti-ZnO NPs.

**Figure 3:**
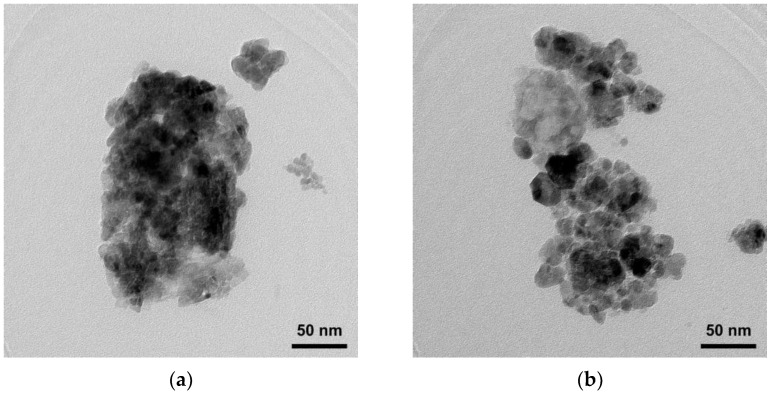
TEM images of (**a**) ZnO and (**b**) Ti-ZnO NPs.

**Figure 4:**
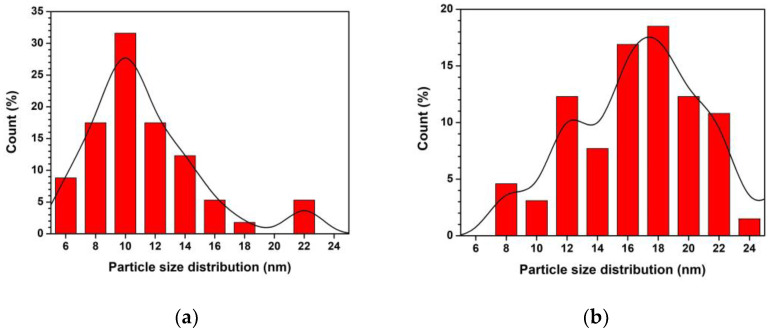
Particle size distribution of (**a**) ZnO and (**b**) Ti-ZnO NPs.

SEM images of ZnO and Ti-ZnO (Figure 2) were observed for the morphological investigation. The morphology showed ZnO and Ti-ZnO nanoparticles (NPs). It seems that numerous accumulated and dense NPs formed cluster-like structures, which is consistent with the TEM images in Figure 3. It is believed that the rapid combustion process provides for fast and disordered crystallization. The process might limit the reaction between zinc and hydroxide ions during the rapid combustion for a short duration, resulting in a randomized reaction that results in clustered NPs [24]. The particle size was estimated using image-J software, as depicted in Figure 4. The maximum particle size distribution for ZnO and Ti-ZnO was found to be 10 nm and 18 nm, respectively, indicating their small size. To examine the chemical elements, EDS results were tabulated in Table 1, indicating the detection of Zn, O, and Ti. The detected elements revealed only the presence of Zn and O for ZnO. In the case of Ti-ZnO, adding the Ti element indicated the presence of Ti in Ti-ZnO structures.

**Figure 5:**
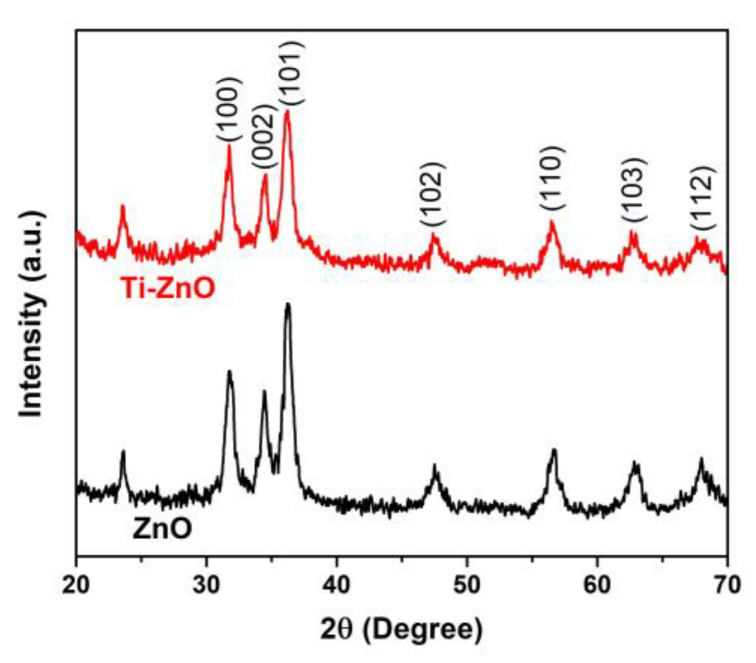
XRD patterns of ZnO and Ti-ZnO NPs.

**Figure 6:**
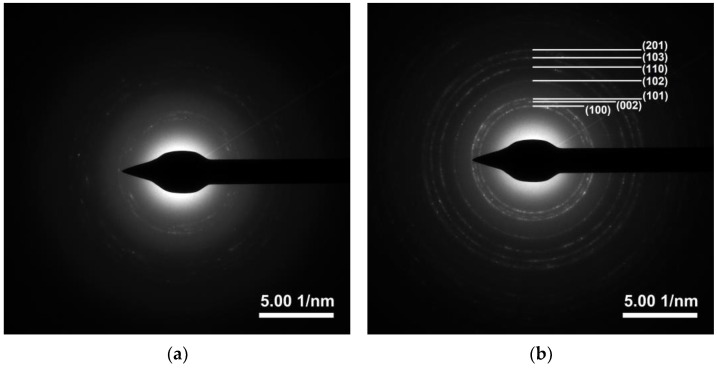
SAED patterns of (**a**) ZnO and (**b**) Ti-ZnO NPs.

The XRD patterns of ZnO and Ti-ZnO in Figure 5 show the diffraction angles at 2θ of 31.7°, 34.5°, 36.2°, 47.5°, 56.6°, 62.9°, and 68.0° indexed to the crystal planes of (100), (002), (101), (102), (110), (103), and (122), respectively [22,25]. The patterns corresponded well with the hexagonal wurtzite structure of ZnO, according to the standard JCPDS no. 36-1451 [2]. The peak at 2θ~23.5° might be related to the orthorhombic Zn(OH)_2_ phase (JCPDS no. 01-071-2215). To estimate the crystal sizes of ZnO and Ti-ZnO, the three major peaks, including the (100), (002), and (101) planes, were considered for the calculation using the Debye–Scherrer equation (Equation (1)). The calculated crystal sizes were 12.88 ± 2.33 and 12.81 ± 2.88 nm for ZnO and Ti-ZnO, respectively. The results presented no differences in the average crystal size for ZnO and Ti-ZnO. This can indicate that Ti^4+^ ions can substitute into Zn^2+^ sites, because the ionic radius of 0.61 Å for Ti^4+^ ions is lower than that of 0.74 Å for Zn^2+^ ions [26]. However, the change in crystal size was not significant for ZnO and Ti-ZnO due to employing the rapid combustion synthesis method. The SAED patterns (Figure 6) displayed the ring patterns for both ZnO and Ti-ZnO implying the polycrystalline structure. This was caused by the limited crystallization due to the short combustion reaction, which caused low crystallization rates and an uncontrollable crystal growth mechanism.

**Figure 7:**
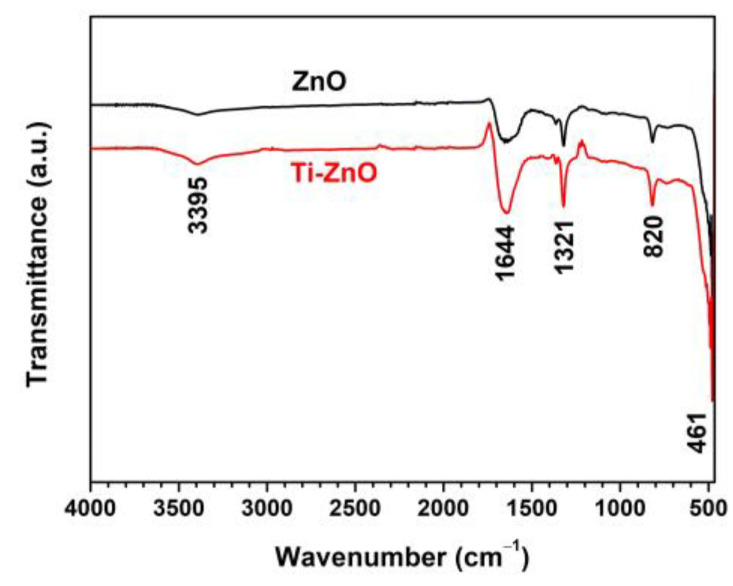
FTIR spectra of ZnO and Ti-ZnO NPs for surface chemical structure analysis.

Figure 7 shows the FTIR spectra of ZnO and Ti-ZnO in the range of 4000–450 cm^−1^ for the examination of the surface chemical structures. The peak at around 461 cm^−1^, referring to the Zn−O vibration, confirmed the ZnO structure [26,27]. The other peaks were marked as residuals, including organic compounds and water. The peaks at 1321 and 1644 cm^−1^ were assigned to C−O stretching and C=O stretching vibrations, respectively [22]. The peak at 820 cm^−1^ was attributed to the OH libration, caused by the Zn(OH)_2_ phase, and it was consistent with the impurity peak in the XRD results. The peak at 3395 cm^−1^ was assigned to the stretching vibration of the OH groups.

**Figure 8:**
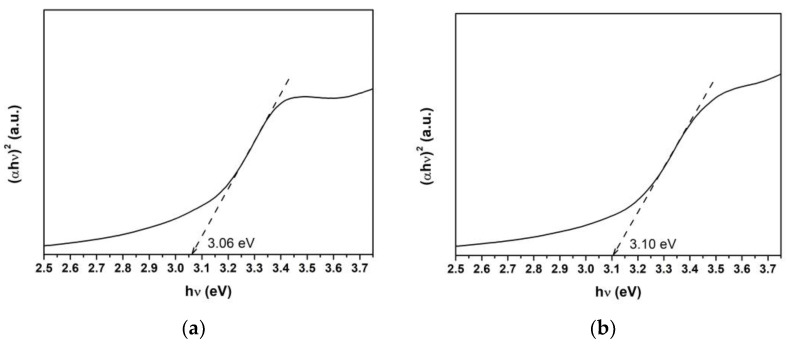
Band gap energy estimation using the Tauc plot for (**a**) ZnO and (**b**) Ti-ZnO.

The band gap energy (E_g_) for the combustion-synthesized ZnO and Ti-ZnO samples was assessed using the Tauc’s plot relation, as shown in Figure 8. For this purpose, a linear line with the maximum slope was extrapolated to the hν-axis. Then, the E_g_ value was estimated from the intercept on the hν-axis. It was found that the E_g_ values for ZnO and Ti-ZnO were 3.06 and 3.10 eV, respectively. However, the E_g_ values exhibited small differences; the obtained values in this study were lower than those in the literature [2,9,12,28,29], which is due to the low crystallization and the defects [20]. The estimated E_g_ values were correlated with the UV energy, indicating that ZnO and Ti-ZnO can be used as photocatalysts and activated by UV light.

**Figure 9:**
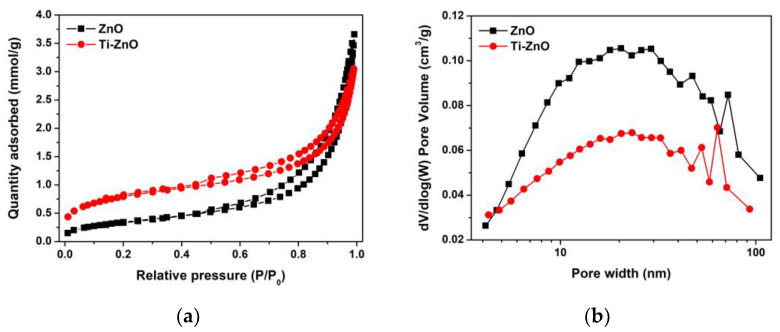
(**a**) N_2_ adsorption–desorption and (**b**) pore width distribution for ZnO and Ti-ZnO NPs.

The N_2_ adsorption–desorption was recorded (Figure 9a) to examine the pore characteristics. The type IV isotherm was similarly observed for both ZnO and Ti-ZnO indicating the mesoporous structure. The H3 hysteresis loop was observed, implying the non-rigid aggregation structures that confirm the cluster-like structure of ZnO and Ti-ZnO. In Figure 9b, the pore width shows a similar pore width distribution. However, the pore widths of 14.75 and 9.12 nm for ZnO and Ti-ZnO, respectively, were estimated using the BJH analysis. S_BET_ was then calculated for the type IV isotherms to estimate the active surface areas of ZnO and Ti-ZnO photocatalysts. The calculated S_BET_ values of 27.84 and 63.85 m^2^/g for ZnO and Ti-ZnO, respectively, were achieved. The results indicate that Ti-ZnO has a greater active surface area than that of ZnO by over two times, which confirms its suitability for photocatalytic applications.

**Figure 10:**
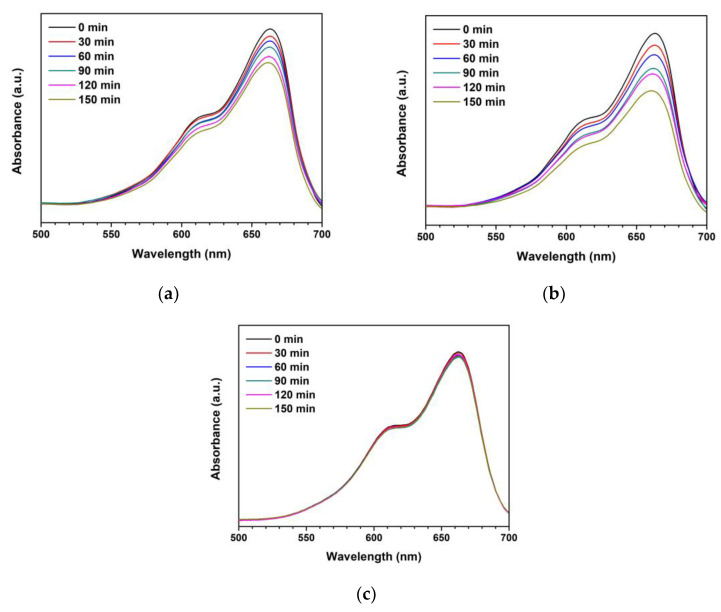
Absorbances of MB under photocatalytic degradation for different UV irradiation time using (**a**) ZnO photocatalyst, (**b**) Ti-ZnO photocatalyst, and (**c**) no photocatalyst.

**Figure 11:**
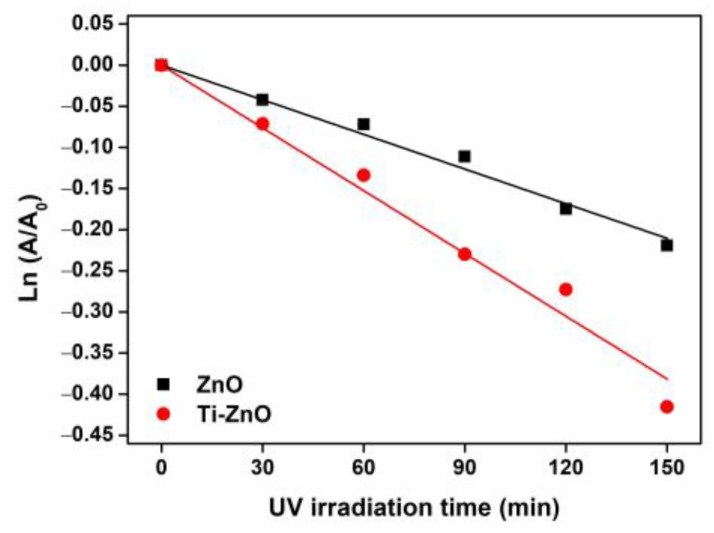
Plots of ln (A/A_0_) versus UV irradiation time for analysis of degradation rate constant.

The synthesized ZnO and Ti-ZnO were used as the photocatalysts for photocatalytic degradation of MB under UV activation. The degradation of MB using only a photocatalyst was examined, as shown in Figure S1 (Supplementary Materials). The negligible change in absorbance of MB demonstrates that the photocatalyst had no significant effect on MB without UV light. To further investigate the influence of photocatalytic activity on MB, the experiment was repeated using ZnO and Ti-ZnO photocatalysts under UV light. The absorbance of MB was measured to investigate the degradation performance, as shown in Figure 10. It was observed that the absorbance of MB for both ZnO and Ti-ZnO photocatalysts decreased as a function of the UV irradiation time. The absorbance of MB for Ti-ZnO showed stronger decreasing trends than that for ZnO. This indicates a better photocatalytic activity for Ti-ZnO to degrade MB molecules compared with ZnO. This is correlated with the higher S_BET_ value for Ti-ZnO. It is believed that the high surface areas can provide interaction between electron-hole carriers and surrounding molecules [21,30], whereas, the unchanged absorbance of MB after UV irradiation in the absence of a photocatalyst confirms that the UV light had no effect on MB. In the analysis of MB degradation performance, the degradation rate constant was estimated using pseudo-first-order kinetics. From Equation (3), the ln (A/A_0_) curve was plotted versus the irradiation time (Figure 11), and the degradation rate constant was calculated by tracking the slopes of the curve. The degradation rate constant values of MB degradation were 1.40 × 10^−3^ and 2.54 × 10^−3^ min^−1^ for ZnO and Ti-ZnO photocatalysts, respectively. The results confirm that Ti-ZnO prepared by combustion has efficient photocatalytic activity for MB degradation, which is consistent with Ti-ZnO prepared by chemical precipitation as described elsewhere [30]. Remarkably, the degradation rate constant for Ti-ZnO increased by over 80% in comparison with ZnO. This result implies that Ti-ZnO improved the kinetic activity of MB degradation under UV illumination. The improved activity is believed to be due to the increased surface area and the reduced pore width. The mechanism of photocatalytic degradation can be briefly described in Equations (4)–(7) [2,31,32]:

ZnO + hν → e^−^ + h^+^ (4)

(5) $$e^{-}+O_{2}\;\to \;O_{2}^{\cdot -}$$

(6) $$h^{+}+H_{2}O\;\to \;{\mathrm{OH}}^{\cdot -}+H^{+}$$

(7) $$\mathrm{Dyes}+O_{2}^{\cdot -}\;\mathrm{or}\;{\mathrm{OH}}^{\cdot -}\;\to \;\mathrm{intermediates}\;\to \;{\mathrm{CO}}_{2}+H_{2}O+{\mathrm{NH}}_{4}^{+}+{\mathrm{SO}}_{4}^{2-}$$

**Figure 12:**
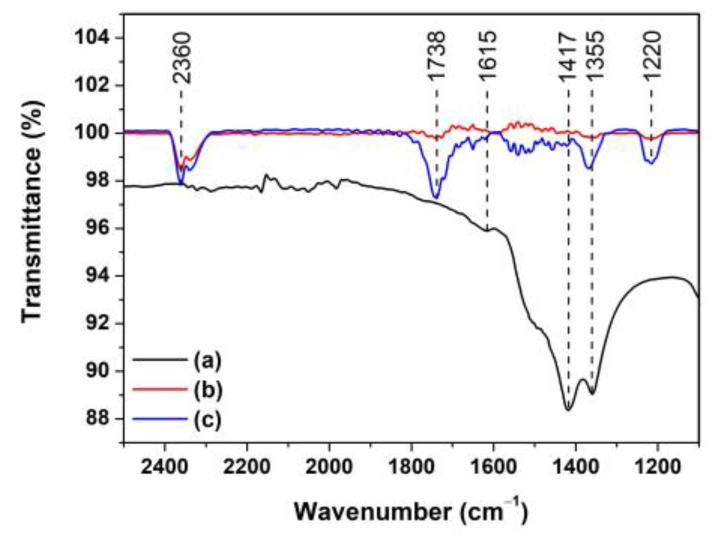
FTIR spectra of (**a**) initial MB solution; and MB solution after photocatalytic degradation for 150 min using (**b**) ZnO and (**c**) Ti-ZnO photocatalysts.

The electron-hole (e^−^-h^+^) pairs are simultaneously generated as the photocatalyst is activated by absorbing photons. They also continuously diffuse onto the photocatalyst surface [33]. The reaction between the electrons and dissolved oxygen (O_2_) in the MB solution induces superoxide radicals ($O_{2}^{\cdot -}$). In another reaction, the holes react with water to produce hydroxyl radicals (${\mathrm{OH}}^{\cdot -}$). These reactive radicals react efficiently to degrade MB dyes into other products. To examine the products, in this study, the MB solution was monitored before and after the photocatalytic degradation for 150 min using ZnO and Ti-ZnO (Figure 12). The initial MB solution peaks were observed at the wavenumber of 1615, 1417, and 1355 cm^−1^ corresponding to the C=N stretching of the central ring, the multiple rings stretching, and C−N stretching of the side aromatic ring and a nitrogen atom, respectively [30]. The intensity of the peaks decreased after photocatalytic degradation. This was attributed to the transformation of MB molecules into intermediates. Moreover, the presence of peaks at 2360, 1738, and 1220 cm^−1^ was assigned to the C=O asymmetric stretching of CO_2_, C=O stretching of aldehyde, and S−O stretching of sulfate ions (${\mathrm{SO}}_{4}^{2-}$) [34,35], respectively. This indicates the conversion of MB into other products. Furthermore, the intensity of the CO_2_ peak for the Ti-ZnO photocatalyst was higher than that of the ZnO. It can be concluded that the high surface area of Ti-ZnO plays a role in offering active sites for the reaction between electron-hole pairs and MB molecules. This effect leads to accelerating the degradation mechanism and raising the degradation performance.

## 4. Conclusions

ZnO and Ti-ZnO NPs were synthesized using rapid combustion. The ZnO and Ti-ZnO structures exhibited similar structures and sizes of polycrystalline hexagonal wurtzite structures, which was due to the substitution of Zn^2+^ sites in the ZnO host lattice by Ti^4+^. The chemical structure examination implied the major vibrations of the ZnO structure. However, some residuals, including organic compounds and water, were also observed. The N_2_ adsorption–desorption analysis confirmed the mesoporous and non-rigid aggregation structures for both ZnO and Ti-ZnO. However, Ti-ZnO showed a higher surface area than that of ZnO by more than double. This is the major factor in improving the photocatalytic activity of MB degradation. The photocatalytic degradation analysis showed the kinetic degradation rate constant of 2.54 × 10^−3^ min^−1^ for Ti-ZnO, which was higher than that of 1.40 × 10^−3^ min^−1^ for ZnO by over 80%. The transformation mechanism of MB molecules into other products, including carbon dioxide, aldehyde, and sulfate ions, was also interpreted in this study.

## Figures and Tables

**Table 1:** Detected chemical composition of ZnO and Ti-ZnO using EDS.

Sample	Element (Atomic%)		
	Zn	O	Ti
ZnO	42.97	57.03	-
Ti-ZnO	33.43	66.05	0.51

## Data Availability

Not applicable.

## Supplementary Materials

The following supporting information can be downloaded at: https://www.mdpi.com/article/10.3390/toxics11010033/s1, Figure S1: Absorbances of MB in the dark condition with the dispersion of (a) ZnO and (b) Ti-ZnO photocatalysts.
