# Supplementary material for: Enhanced Photocatalytic Degradation of Methylene Blue Using Ti-Doped ZnO Nanoparticles Synthesized by Rapid Combustion
Source: Toxics. 2022 Dec 29;11(1):33. doi: 10.3390/toxics11010033 (PMC9865418; doi:10.3390/toxics11010033)
Supplement: Supplementary file 1 [file toxics-11-00033-s001.zip › toxics-2113215-supplementary.pdf]

toxics

# Enhanced Photocatalytic Degradation of Methylene Blue Using Ti-Doped ZnO Nanoparticles Synthesized by Rapid Combustion

Sutthipoj Wongrerkdee, Sawitree Wongrerkdee, Chatdanai Boonruang, Supphadate Sujinnapram

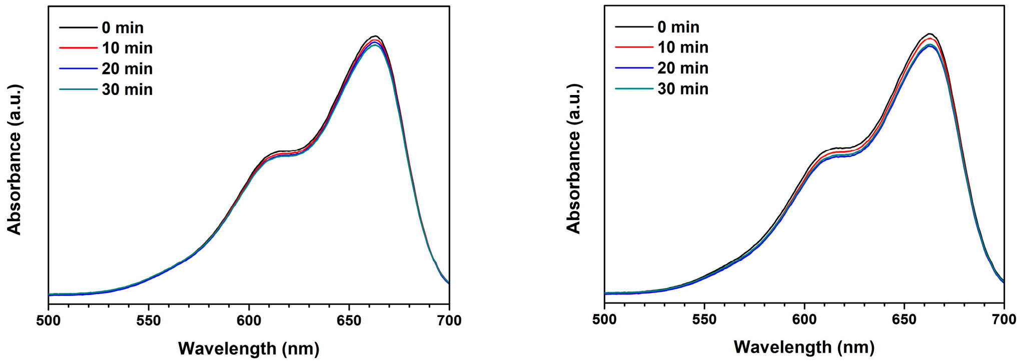


(**a**) (**b**)

**Figure S1.** Absorbances of MB in the dark condition with the dispersion of (a) ZnO and (b) Ti-ZnO photocatalysts.
